# Decreased bilateral thalamic gray matter volume in first-episode schizophrenia with prominent hallucinatory symptoms: A volumetric MRI study

**DOI:** 10.1038/srep14505

**Published:** 2015-09-25

**Authors:** Peng Huang, Yibin Xi, Zhong-Lin Lu, Yunchun Chen, Xiangrui Li, Weiguo Li, Xia Zhu, Long-Biao Cui, Qingrong Tan, Wenming Liu, Chen Li, Danmin Miao, Hong Yin

**Affiliations:** 1 Department of Medical Psychology, The Fourth Military Medical University, Xi’an, Shaanxi 710032, China; 2 Department of Radiology, Xijing Hospital, The Fourth Military Medical University, Xi’an, Shaanxi 710032, China; 3 The Center for Cognitive and Behavioral Brain Imaging (CCBBI), Department of Psychology, The Ohio State University, Columbus, OH 43210, USA; 4 Department of Psychiatry, Xijing Hospital, The Fourth Military Medical University, Xi’an, Shaanxi 710032, China; 5 Department of Bioengineering, University of Illinois at Chicago, Chicago, IL 60612, USA

## Abstract

Studies comparing gray matter (GM) volume of schizophrenic patients with or without auditory verbal hallucinations (AVHs) to that of normal controls remain controversial. This project aims to investigate changes of GM volumes of drug-naïve schizophrenic patients with and without AVHs. Eighteen first episode schizophrenic (FES) patients with AVHs, 18 FES patients without AVHs, and 18 healthy controls were scanned using structural MRI. Voxel-based morphometry (VBM) analysis was conducted to investigate changes of GM volume among the three groups. Patients with and without AVHs exhibited reduced GM volumes relative to normal controls in the left superior temporal gyrus, frontal regions, cerebellum and caudate. Further analysis of the GM of subcortical structures found that patients with AVHs had reduced thalamic volume than healthy controls. No significant difference was found between patients with and without AVHs. Significant correlation was found between the total scores of the Positive and Negative Syndrome Scale and bilateral thalamic volume. ROC analysis of thalamic volumes of the patients with AVHs and normal controls showed that the area under the curve was 0.698 (*P* = 0.043). The decreased thalamic volumes might serve as a biomarker for discriminating FES AVHs patients from normals.

Schizophrenia is a severe and disabling brain disorder. Nearly 60–80% of schizophrenic patients are liable to experience auditory verbal hallucinations (AVHs)^1^,^2^. It has been reported that about one fourth of patients with AVHs are chronic and resistant to medication^3^.

Abnormalities of brain structures have been well established in schizophrenia^4^,^5^. However, the reported nature of abnormality has been inconsistent. On the one hand, neuroimaging studies on FES patients have found reduced gray matter volume in several cortical regions compared to normal controls. For example, using the region-of-interest (ROI) approach, studies on adolescent schizophrenia found reduced volume in the ventricle system, the superior temporal gyrus (STG), and the thalamus^6^,^7^. Reduced thalamic and nucleus accumben volumes in FES patients have also been reported^8^,^9^. On the other hand, some longitudinal studies found no volumetric difference of any brain structure between patients with schizophrenia and healthy controls^10^. Several meta-analyses^7^,^11^ supported brain volume changes in the frontal, temporal and parietal regions. The inconsistency might be due to the complex pathogenesis and long-term chronicity of the disease and different methodological approaches of these studies^6^. The lack of a clear understanding of auditory hallucinations^12^ may also contribute to the inconsistency. In the current drug-naïve first-episode schizophrenia study, evaluating a specific subgroup of schizophrenia, i.e., those with AVHs, may allow us to reduce the underlying neural variability and resolve some of the inconsistencies^13^. In a schizophrenia mouse model, Chun *et al*. have demonstrated that specific disruptions of the thalamic-auditory cortical pathway altered auditory information processing and led to AVHs when the animals were put under stress or other confounding conditions^14^.

For structural brain analysis, the whole brain VBM has many advantages over the ROI approach. It is automated rather than observer-based^15^; it covers the whole brain and involves less laborious processing^16^. In this study, we conducted a whole brain VBM analysis to investigate GM volume changes in FES patients with and without AVHs. We hypothesize that GM volume in a few brain regions, including the left superior temporal gyrus, inferior frontal gyrus (IFG), and thalamus, is reduced in FES patients with AVHs compared to normal controls.

## Results

### Demographic and clinical-scale characteristics of the subjects

The demographic and clinical-scale characteristics of the patients and normal controls are listed in Table 1. There are obvious differences in education level and PANSS positive and total scores, but the three groups do not differ significantly in terms of age and gender.

### Voxel-based morphometry analysis of gray matter volume

**Figure 1:**
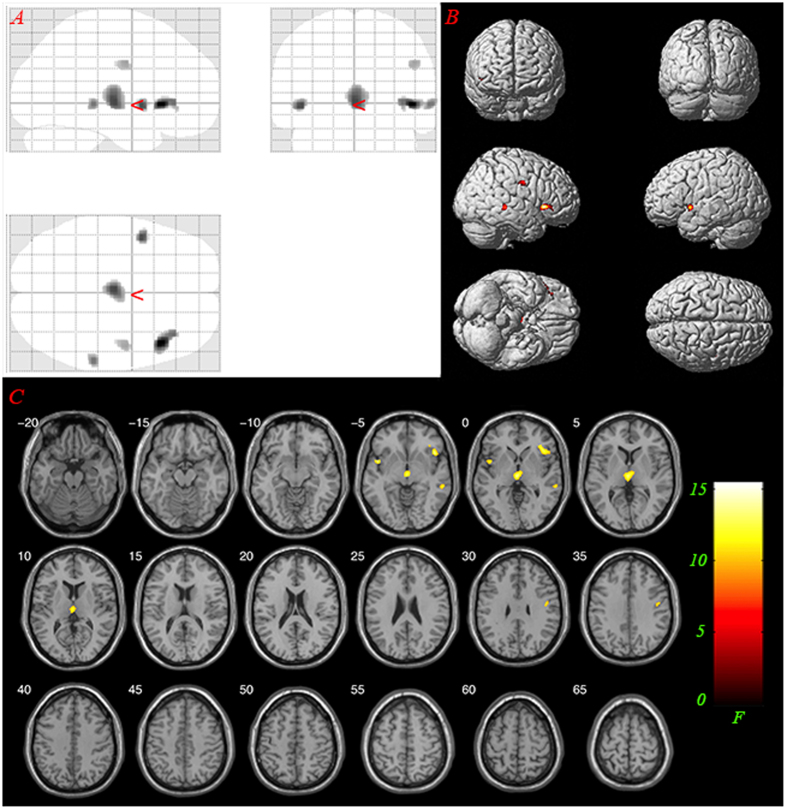
Main effects: significant GM differences in the bilateral thalamus, left Superior Temporal Gyrus, right Inferior Frontal Gyrus, Inferior Parietal Lobule and Precentral Gyrus (*F*_(2, 48)_ = 8.004, *P* < 0.001 uncorrected, cluster size >65). (**A**) glass view; (**B**) 3-D render; (**C**) slice view. Only clusters thresholded with the number of expected voxels are shown.

**Figure 2:**
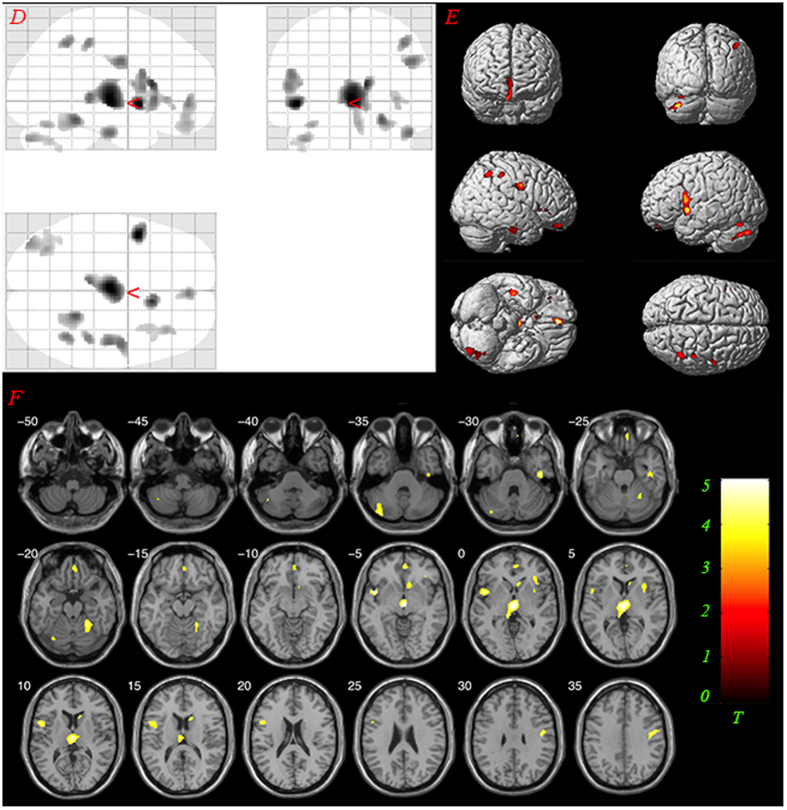
Results of post hoc analysis comparing GM volumes between groups AVHs and NC. The yellow-colored areas indicate brain regions, including bilateral thalamus, left Superior Temporal Gyrus, left Inferior Frontal Gyrus, right Inferior Parietal Lobule, Inferior Temporal Gyrus, Cerebellum Posterior Lobe, Cerebellum Anterior Lobe, Caudate, Medial Orbital Frontal Gyrus, and Precentral Gyrus, with significantly reduced GM volumes relative to normal controls. The statistical threshold was set at *P* < 0.001 uncorrected. (AVHs: auditory verbal hallucinations; NC: normal controls). (**D**) glass view; (**E**) 3-D render; (**F**) slice view. Only clusters thresholded with the number of expected voxels are shown.

**Figure 3:**
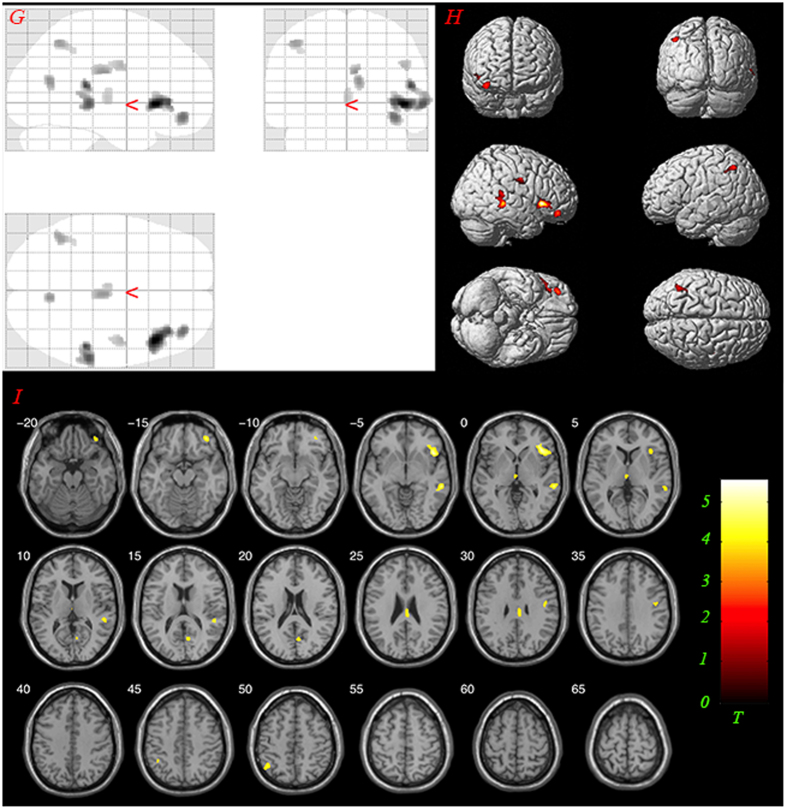
Results of post hoc analysis comparing GM volumes between groups Non-AVHs and NC. The yellow-colored areas indicate brain regions, including left thalamus, right Inferior Frontal Gyrus, left Inferior Parietal Lobule, Middle Temporal Gyrus, Middle Orbital Frontal Gyrus, with significantly reduced GM volumes relative to normal controls. The statistical threshold was set at *P* < 0.001 uncorrected. (AVHs: auditory verbal hallucinations; NC: normal controls). (**G**) glass view; (**H**) 3-D render; (**I**) slice view. Only clusters thresholded with the number of expected voxels are shown.

Voxel-based morphometry (VBM) analysis and one-way ANOVA were conducted. Difference in GM volume was found in the bilateral thalamus, left STG, right IFG and precentral gyrus among the three groups (*F*_(2, 48)_ = 8.004, *P* < 0.001 uncorrected, cluster size >65 (expected voxels per cluster). See details in Fig. 1). Post-hoc analysis showed that the gray matter volumes of the patients with AVHs was significantly reduced in the bilateral thalamus, left STG, left IFG and inferior parietal lobule, inferior temporal gyrus, cerebellum posterior lobe, cerebellum anterior lobe, caudate, medial orbital frontal gyrus, and precentral gyrus compared to those of normal controls (*T* = 3.268, *P* < 0.001 uncorrected, cluster size >95 (expected voxels per cluster). See details in Fig. 2). Post-hoc analysis also showed that the gray matter volumes of the patients without AVHs was significantly reduced in the left thalamus, IFG, inferior parietal lobule, middle orbital frontal gyrus and middle temporal gyrus compared to those of normal controls (*T* = 3.268, *P* < 0.001 uncorrected, cluster size >95 (expected voxels per cluster). See details in Fig. 3). Detailed results are provided in Figs 1,2,3 and Table 2.

### Comparison of thalamic volumes between patients and normal controls

**Figure 4:**
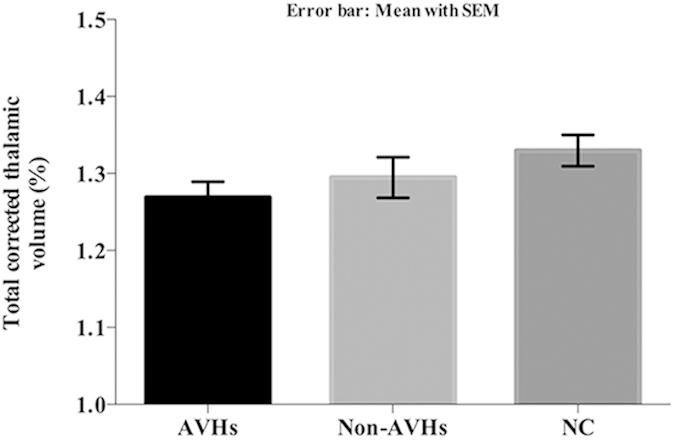
Comparison of corrected thalamic volumes between the two patient and normal control groups. Significant difference was found between groups AVHs and group NC. (AVHs: Schizophrenic patients with auditory verbal hallucinations; Non-AVHs: Schizophrenic patients without auditory verbal hallucinations; NC: Normal controls).

Each subjects’ bilateral thalamic volumes were corrected for differences in whole brain size to eliminate potential head size bias. Age, gender, and education years were considered as covariates as well. Thalamic volumes decreased from normal controls (group NC, 1.330 ± 0.086%) to patients without AVHs (group Non-AVHs, 1.295 ± 0.112%) and patients with AVHs (group AVHs, 1.269 ± 0.086%). We found that the group factor (F_(2, 47)_ = 3.284 , *P* = 0.046) had significant impact on thalamus volume. Further post-hoc analysis (Least Significant Difference, LSD) showed that, significance difference was found between groups NC and AVHs (*P* = 0.014). The results are shown in Fig. 4. No significant difference was found between groups AVHs and Non-AVHs, nor between groups Non-AVHs and NC.

### Correlation between thalamic volumes and clinical scale scores

**Figure 5:**
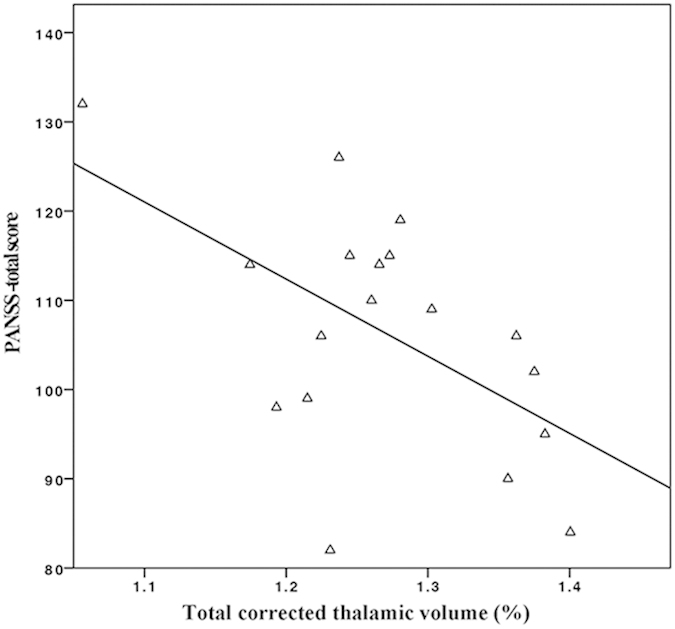
The PANSS total scores were significantly correlated with the corrected bilateral thalamic volumes in the group AVHs (PANSS: Positive and Negative Syndrome Scale, AVHs: Schizophrenic patients with auditory verbal hallucinations, r = −0.551, *P* = 0.018).

We found a significant correlation between the PANSS total scores and corrected bilateral thalamic volumes in the AVHs group (r = −0.551, *P* = 0.018, see Fig. 5). HAHRS scores were obtained for patients with AVHs. We did not find a significant correlation between the HAHRS scores and corrected bilateral thalamic volumes in the group (r = −0.149, *P* = 0.555).

### ROC analysis of thalamic volumes of patients with AVHs and normal controls

**Figure 6:**
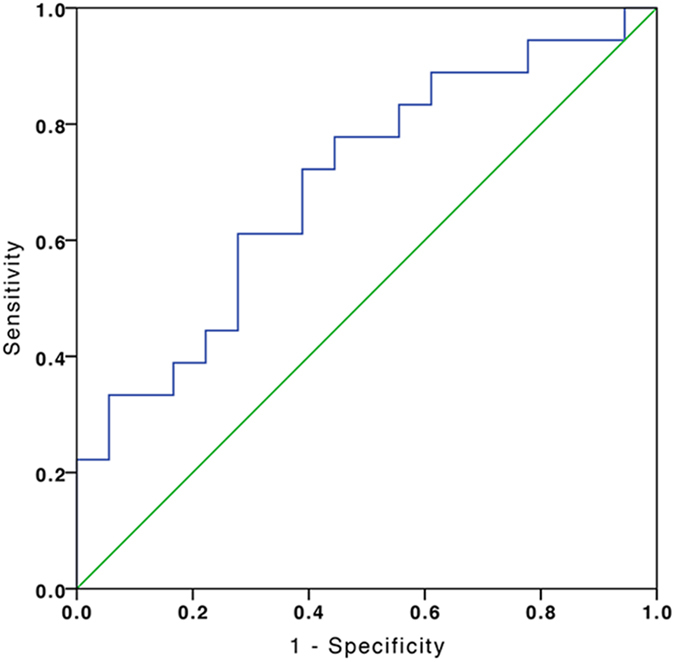
ROC curve for discriminating schizophrenic patients with AVHs from normal controls. The area under the ROC curve was 0.698 (*P* = 0.043). ROC: Receiver Operating Characteristic; AVHs: Schizophrenic patients with auditory verbal hallucinations.

A receiver operating characteristics (ROC) graph is a technique for visualizing, organizing and selecting classifiers based on their performance^17^. ROC analysis, which has been widely used in the evaluation of diagnostic tests^18^, was performed on the corrected thalamic volume to classify patients with AVHs and normal controls into their respective categories. We found that all the corrected thalamic volumes were located within Mean ± 3SD in the NC and AVHs groups. Grubbs test^19^ indicates that there is no outlier in the dataset (α = 0.05). The area under the ROC was 0.698 (*P* = 0.043). According to Youden’s index^20^, when the corrected thalamic volume is greater than 1.267%, the corresponding sensitivity and specificity of the ROC analysis was 77.8% and 55.6% respectively (see Fig. 6).

## Discussion

In the current study, we found that the GM volumes of a number of brain structures, including the bilateral thalamus, left superior temporal gyrus (STG), frontal regions, precentral gyrus, cerebellum and caudate, were significantly reduced in drug-naïve FES patients with AVHs, compared to those of normal controls. There were significant GM volume differences of GM in left thalamus, IFG, inferior parietal lobule, middle orbital frontal gyrus and middle temporal gyrus, between the FES patients without AVHs and normal controls. No significant difference was found between FES patients with and without AVHs.

The STG is involved in speech perception^12^. Our finding that GM volume of the left STG is reduced in schizophrenic patients with AVHs is consistent with that of Modinos and colleagues^21^ who found that the level of AVHs was significantly correlated with GM volume reduction in the left STG in a meta-analysis. The left STG includes the auditory cortex and has been shown to be the aetiology of AVHs^22^. All subjects were right-handed in our study. It is conceivable that the left STG may play a critical role in AVHs. Kubera *et al*. has found that the GM volume of the frontotemporal region was reduced in persistent AVHs patients^23^. We have found reduced GM volume in the left inferior frontal gyrus (Brodmann’s areas 44 and 45), medial frontal gyrus (Brodmann’s areas 8, 11). The left inferior frontal gyrus is one of the classical left-hemisphere language-processing areas^24^; the medial frontal gyrus is associated with language and memory processing^25^,^26^. A number of studies have suggested that the cerebellum is involved in schizophrenia^27^,^28^. For instance, a significant reduction of the volume of the cerebellum vermis in the schizophrenic group has been reported^29^, and Neckelmann *et al.*^30^ found that the severity of hallucinations associated with reduced grey matter volume in left and right cerebellum. In the current study, we found the reduced cerebellum volume in the AVHs group compared to normal controls. The cerebellum is thought to be involved in the monitoring process^22^. However, conventional VBM method lacks some precision with respect to cerebellar morphology, so we would treat this conclusion with care. The structural studies, e.g., gray matter volume changes in caudate^31^ and precentral gyrus^32^, have shown that the caudate and precentral gyrus were associated with hallucination or inner speech.

In this study, we found no significant correlation between scores of the auditory hallucination rating scale and thalamic volume in the AVHs group. But there was a significant correlation between PANSS total scores and thalamic volume in the AVHs group. Our ROC analysis suggests that it was possible to use thalamic volume to discriminate schizophrenic patients with AVHs from normal controls with modest success.

There are several limitations in this study. First, we only conducted a VBM analysis on the data. Future research could apply source-based morphometry (SBM)^33^ on the dataset. Second, our sample size is relatively small due to the difficulty in recruiting drug-naïve first-episode schizophrenic patients with auditory verbal hallucinations.

Similar to many studies that have found reduced thalamic volume in patients with schizophrenia^34^,^35^, we found significant GM volume differences in brain structures between first-episode schizophrenic patients with or without hallucinations and normal controls. To our knowledge, the findings that there’s no volumetric difference in drug-naïve first-episode schizophrenia with AVHs versus Non-AVHs is a novel result. In fact, there’s just one study focusing on volumetric differences in drug- naïve AVHs versus Non-AVHs. In that study^36^, the authors found that the gray matter volumes in the frontal and temporal lobes were significantly larger in patients with AVHs than in patients without AVHs. The different results from the two studies are probably due to (1) smaller sample size (17 AVHs, 8 Non-AVHs), (2) no adjustment on sex, and (3) unbalanced number of patients with and without AVHs. The negative findings between AVHs group and Non-AVHs group patients may be because two of our Non-AVHs patients have reported that they’ve experienced AVHs 2 years prior to recruitment. We have excluded these two patients in the Non-AVHs group, and did the VBM again, the results were similar to the current study and the difference between two patients group is still negative.

As a debilitating and often lifelong disease^37^, schizophrenia is considered to be a nosology that likely reflects a group of diseases rather than a single disease entity^38^,^39^. The substantial clinical heterogeneity in this disorder is a major obstacle to the identification of the neurobiological correlates of this disorder^40^. In this study, the FES patients were young and didn’t have long duration of the disease. Guo *et al*. found that patients with longer psychosis histories had significantly smaller gray matter volumes in the right superior temporal gyrus, left fusiform gyrus, and left middle temporal gyrus^41^. But no such significant effect was found in this study. Although a consensual definition is still lacking, AVHs is characterized by perceiving sounds without auditory stimulus, there’s one of the most influential cognitive models proposes the impairment of self-monitoring on one’s own inner speech, then misleading to identify verbal thoughts as alien source^42^,^43^,^44^. There is evidence that AVHs could act as a potential taxonomy standard^45^. That’s probably why we found significant difference between patients with AVHs and normal controls, especially in the left STG. In order to reduce or eliminate clinical heterogeneity in a study sample, an endophenotype (intermediate phenotype) research strategy should be adopted^45^. An important requirement, however, is that an endophenotype should be a quantitatively measurable trait.

## Conclusion

In conclusion, the current study demonstrates that volumetric reductions in language processing areas and nonsensory regions in FES patients with AVHs. Significant reduction in thalamic volume, especially the right thalamic volume, may provide an objective imaging measure for discriminating schizophrenic patients with auditory verbal hallucinations from normal controls.

## Methods

### Participants

The included 36 FES patients were assigned to two groups according to the presence of AVHs symptom. Those who reported AVHs at least once a day for the past four weeks were assigned to AVHs group. Patients who have never experienced AVHs or have not experienced them within two years before recruitment were allocated to Non-AVHs group. An additional 18 normal control subjects (NC group), matched for age and gender with the patients, were recruited via advertisement in the local community. All participants gave their written informed consent prior to the study, which was in accordance with the Declaration of Helsinki and all experiments protocol were approved by ethics committee in Fourth Military Medical University, Xi’an, China.

### Clinical measures

Patients meeting diagnostic criteria for schizophrenia according to DSM-IV were assessed by two senior clinical psychiatrists using the Positive and Negative Symptom Scale^46^ (PANSS total score ≥60) at most two days before the MRI session. All patients were first-episode and drug-naïve at the time of scanning. AVHs patients were further evaluated using Hoffman Auditory Hallucination Rating Scale (HAHRS)^47^ before the MRI scan. This scale assesses AVHs on seven characteristics: frequency, reality, loudness, number of voices, length, attention dedicated to the hallucinations, and hallucination-induced arousal. A general score is obtained as well, by summing the items, to give a measure of general AVHs severity.

### Magnetic resonance imaging

Magnetic resonance imaging (MRI) was performed on a 3 Tesla Allegra system (Siemens, Erlangen, Germany). A standard birdcage head coil along with foam pads was used to reduce head motion and scanner noise. High-resolution whole brain volume T1-weighted 3D anatomical data were acquired using the 3D magnetization-prepared rapid gradient echo (3D MPRAGE) sequence. The scanning parameters were: repetition time = 2530 ms; echo time = 3.5 ms; flip angle = 7°; field of view = 256 mm × 256 mm; data matrix = 256 × 256; slice thickness = 1 mm; gap = 0 mm; number of slices = 192 slices. The image resolution was 1 mm × 1 mm × 1 mm.

### Image analysis

Data were preprocessed using the VBM8 toolbox (http://dbm.neuro.uni-jena.de/vbm) in the Statistical Parametric Mapping software package version 8 (SPM8; http://www.fil.ion.ucl.ac.uk/spm). The structural T1-weighted anatomical images were automatically segmented into gray matter and white matter. The resulting images were spatially normalized and scaled with Jacobian matrices into the Montreal Neurological Institute (MNI) space. The absolute masking, with a threshold of 0.2 was used. Modulated images were saved by correcting for non-linear warping only. The Gaussian kernel for smoothing had an 8-mm full-width at half maximum (FWHM). Analysis of variance was conducted based on the general linear model. Age, gender, years of education were included as additional covariates in this model. The Xjview toolbox (http://www.alivelearn.net/xjview) was used to perform multi-comparison correction with *P* < 0.001 (uncorrected). Only clusters thresholded with the number of expected voxels (spatial extent threshold) calculated according to the theory of Gaussian random fields are reported.

Individual bilateral thalamic volumes were estimated using FIRST (http://fsl.fmrib.ox.ac.uk/fsl/fslwiki/first), which is part of FSL Version 5.0.6 (FMRIB Software Library) and a model-based segmentation/registration tool for deep gray matter, e.g., left and right thalamus. Total brain volume was estimated with SIENAX (http://fsl.fmrib.ox.ac.uk/fsl/fsl-4.1.9/siena/index.html#sienax), which is a part of FSL^48^. The total volume of brain tissue, normalised for subject head size, was estimated with SIENAX^49^, used to correct thalamic volume for differences in head size.

### Statistical analysis

Chi-square tests, independent-sample t tests, and one-way ANOVAs were conducted in this study. ANOVA and post hoc tests were applied to compare the corrected GM volumes of the three groups. Pearson correlation analysis was applied to evaluate the relationship between thalamic volume and clinical test scores in AVHs patients. A Receiver Operating Characteristic (ROC) analysis was performed to distinguish AVHs patients from normal controls based on thalamic volumes. SPSS 16.0 (IBM SPSS Statistics) was used for additional statistical analysis. *P* < 0.05 was considered statistically significant.

## Additional Information

**How to cite this article**: Huang, P. *et al.* Decreased bilateral thalamic gray matter volume in first-episode schizophrenia with prominent hallucinatory symptoms: A volumetric MRI study. *Sci. Rep.* **5**, 14505; doi: 10.1038/srep14505 (2015).

## Figures and Tables

**Table 1:** Demographic and clinical-scale characteristics of study participants.

	AVHs	Non-AVHs	NC	*P* value
Demographic variables				
Cases	18	18	18	
Age, years (mean ± SD)	22.56 ± 6.73	22.67 ± 3.85	25.06 ± 2.44	0.200(F = 1.63)
Gender (Male/Female)	10/8	9/9	9/9	0.929(χ^2^ = 0.148)
Left/Right Handiness	0/18	0/18	0/18	
Education Level, years (mean ± SD)	12.44 ± 2.30	12.56 ± 2.19	15.44 ± 2.48	0.000(F = 9.72)***
Clinical variables				
Duration of illness, months (mean ± SD)	5.94 ± 5.93	12.44 ± 18.16		0.16(t = −1.44)
PANSS-positive symptoms	31.11 ± 6.92	18.61 ± 8.68		0.006(t = 4.78)**
PANSS-negative symptoms	25.78 ± 3.87	22.06 ± 10.43		0.17(t = 1.42)
PANSS-general psychopathology	49.56 ± 9.01	47.39 ± 9.84		0.50(t = 0.69)
PANSS-total score	106.44 ± 13.55	88.06 ± 23.90		0.008(t = 2.84)**
HAHRS	26.22 ± 8.10			

AVHs: Schizophrenic patients with auditory verbal hallucinations; Non-AVHs: Schizophrenic patients without auditory verbal hallucinations; NC: Normal controls. **P < 0.01, ***P < 0.001.

**Table 2:** Brain clusters with significant gray matter volume changes.

Brain region	L/R	No. of voxels	MNI coordinates (X Y Z)			*P* value
*Main-effect*						*P* < 0.001 uncorrected
Superior Temporal Gyrus	L	67	−45	6	−3	
Thalamus	R	224	4	−16	2	
Thalamus	L	268	−4	−16	6	
Inferior Frontal Gyrus	R	300	45	22.5	−3	
Precentral Gyrus	R	99	46.5	−7.5	31.5	
*Post-hoc (volume reduction in AVHs vs. NC)*						*P* < 0.001 uncorrected
Superior Temporal Gyrus	L	241	−46.5	6	−4.5	
Thalamus	R	448	4.5	−10.5	1.5	
Thalamus	L	651	−1.5	−19.5	1.5	
Inferior Frontal Gyrus	L	143	−49.5	9	19.5	
Inferior Parietal Lobule	R	158	42	−52.5	49.5	
Inferior Temporal Gyrus	R	121	46.5	−16.5	−30	
Precentral Gyrus	R	272	48	−9	32	
Cerebellum Posterior Lobe	L	332	−39	−82.5	−34.5	
Cerebellum Anterior Lobe	R	228	24	−45	−18	
Caudate	R	255	10.5	18	12	
Medial Orbital Frontal Gyrus	R	183	3	48	−3	
*Post-hoc (volume reduction in Non-AVHs vs. NC)*						*P* < 0.001 uncorrected
Thalamus	L	97	−1.5	−18	1.5	
Inferior Frontal Gyrus	R	578	45	21	−3	
Inferior Parietal Lobule	L	144	−43.5	−57	49.5	
Middle Orbital Frontal Gyrus	R	150	37.5	43.5	−13.5	
Middle Temporal Gyrus	R	292	51	−36	13.5	

No. Number; MNI: Montreal Neurological Institute; L/R: Left or Right hemisphere; Cluster size >  expected voxels per cluster; AVHs: Schizophrenic patients with auditory verbal hallucinations; Non-AVHs: Schizophrenic patients without auditory verbal hallucinations; NC: Normal controls.
